# Mediation of mammalian olfactory response by presence of odor-evoked potassium current

**DOI:** 10.3389/falgy.2024.1478529

**Published:** 2024-10-16

**Authors:** Samantha Hagerty, Oleg Pustovyy, Ludmila Globa, Vitaly Vodyanoy, Melissa Singletary

**Affiliations:** ^1^ Department of Anatomy, Physiology and Pharmacology, Auburn University College of Veterinary Medicine, Auburn, AL, United States; ^2^ Canine Performance Sciences Program, Auburn University College of Veterinary Medicine, Auburn, AL, United States

**Keywords:** olfaction, olfactory receptor, G-protein coupled receptor, signal transduction, potassium ion channel, electroolfactogram, electrophysiology

## Abstract

It is well understood that odorants interact with specialized G-protein coupled receptors embedded in the ciliary membrane of olfactory sensory neurons (OSN) which initiates a voltage-generating intracellular cascade of signal transduction events that can be recorded at the epithelial level as an electroolfactogram (EOG). While the depolarizing excitatory pathway in vertebrates involving cyclic adenosine monophosphate (cAMP)-induced Na^+^/Ca^2+^ influx and calcium-induced Cl^−^ efflux is well established, there is evidence of potassium-associated inhibitory currents that correspond with cellular activation. While several Ca^2+^-dependent feedback mechanisms contribute to cellular deactivation which have been commonly attributed to these inhibitory currents, the frequently observed positive ionic conductance prior to excitatory depolarization have led many to suggest an additional earlier inhibitory mechanism at the receptor level that may be independent of downstream calcium influx. Due to conflicting conclusions, the role and mechanism behind Ca^2+^-independent inhibitory currents in olfactory cells is not fully understood. We investigated the functional and temporal involvement of potassium channels in odor transduction by comparing electroolfactogram (EOG) recordings in rat olfactory epithelia following ion channel inhibition and targeted activation of downstream components with or without potassium-blocking. Several K^+^-channel blocking agents (4-Aminopyridine, charybdotoxin, & iberiotoxin) demonstrated a diminished pre-action potential positive current that corresponded with reduced excitatory response to odor stimulation that was recovered when blockers were removed. We further assessed EOG responses in the absence of odor or with odor response enhancing zinc nanoparticles. Chemically eliciting membrane excitation in the absence of odor stimulation with a phosphodiesterase inhibitor, 3-isobutyl-1-methylxanthine (IBMX), in combination with K^+^-channel inhibition, further indicated potassium channel activation precedes excitatory events and is independent of cAMP-induced calcium influx. These results support previous findings of odor-activated inhibitory potassium currents that may play a functional role in subsequent G-protein activity.

## Introduction

While olfaction is the most evolutionarily conserved sensory system across species, several controversial components of signal transduction mechanisms include receptor-odorant interactions, ionic channel activity, and discriminatory coding of the vast array of molecular information into perceived odors. There are, however, many concretely understood concepts of the vertebrate olfactory system that have not wavered since early classical methods of discovery, including morphological characteristics and flow of information along the most identified pathway. The initial process of olfaction occurs in the olfactory epithelium (OE) located in the nasal cavity, where a high population of bipolar olfactory sensory neurons (OSN) reside (1, 2). In most mammals, olfaction begins with the act of sniffing, which transports odorant molecules into the nose and delivers them to the mucus layer covering the olfactory epithelium (3) where they diffuse to the receptor site (4). OSN are positioned transversely in the OE, with a dendritic process ending in many ciliary projections that extend into the mucous layer lining where odorants are dissolved. Cilia are microtubule-based organelles that are essential for olfactory performance. The hair-like ciliary structures protruding apically harbor the sensory apparatus, including the olfactory receptor proteins, heterotrimeric G-proteins, and downstream second messenger components involved in the GPCR cascade (5). In vertebrates, olfactory receptors embedded within the cellular membrane of cilia processes comprise the largest family of G-protein coupled receptors (6). Binding of an odorant molecule to an odorant receptor is thought to cause a conformational change which activates a G-protein complex, stimulating downstream enzymatic activity (7–9). The most well-known intracellular cascade of molecular events involves adenylyl cyclase (AC)-driven cyclic adenosine monophosphate (cAMP) production which activates the opening of cyclic nucleotide gated (CNG) ion channels (10). Na^+^ and Ca^2+^ enter through the opened channel and initiate depolarization of the cell membrane (11–13). The intracellular increase of Ca^2+^ concentration directly opens calcium-activated Cl^−^ ion channels (14, 15), causes anionic efflux of Cl^−^ ions from the cilia, corresponding to the inward positive flow current that amplifies depolarization of OSNs (16, 17). The combined inward positive and outward negative ionic currents generate an overall excitatory generator potential that can be recorded at the cellular surface (18–20). Upon depolarization threshold, an action potential is generated that propagates through a single axon toward the central nervous system (21). The transformation of chemical odorant signal into an electrical signal generates a voltage among the activated population of OSN which can be recorded at the epithelial surface (22, 23).

**Figure 1:**
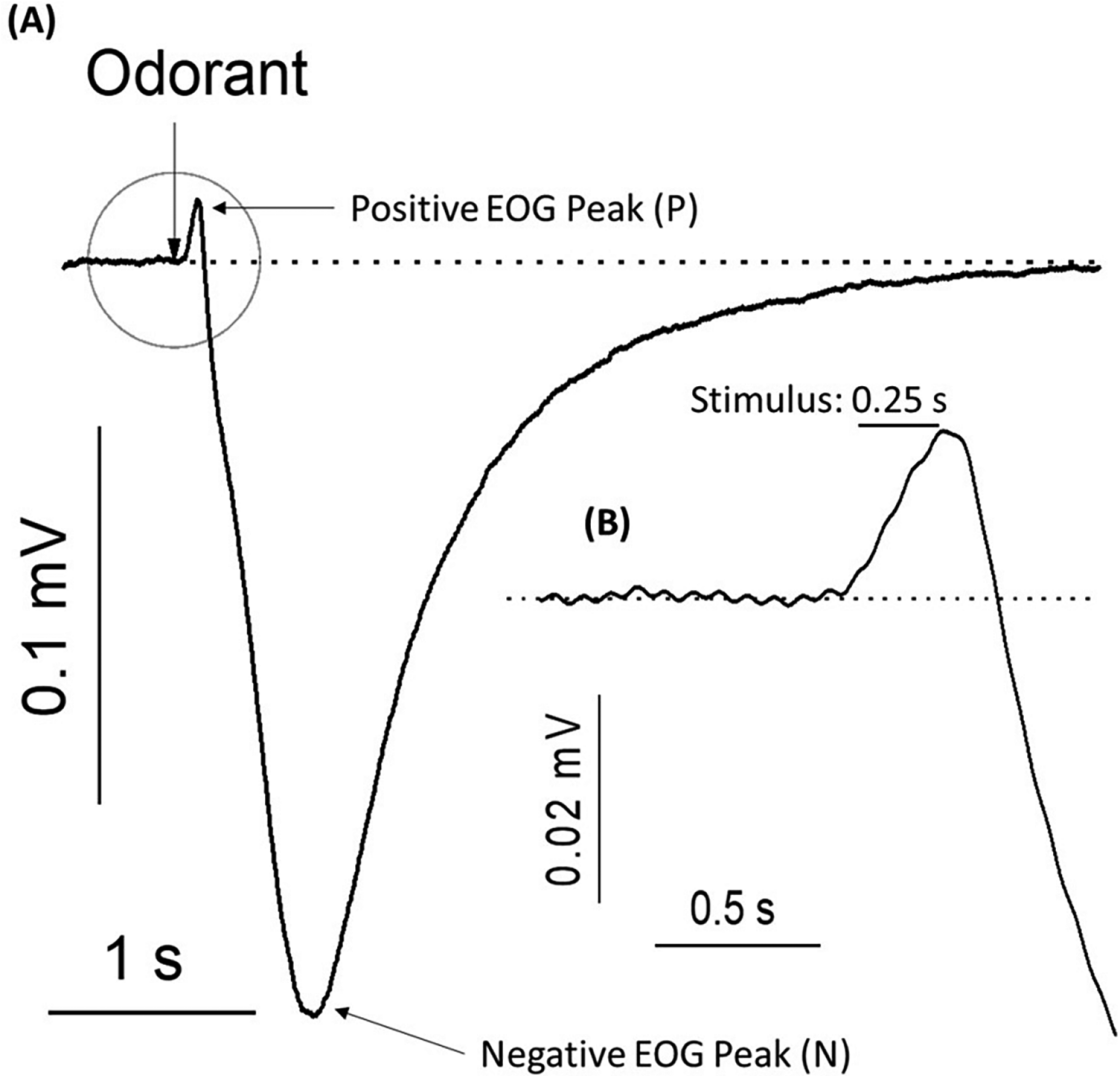
Typical electroolfactogram (EOG) – electrical responses of the olfactory epithelium to the pulse of an odorant. **(A)** Electroolfactogram in a positive direction (P) shows small-amplitude peak after a 0.25-s (0.25 s) pulse of odorant. EOG of the negative direction reaches a maximum Reading (N) before returning to baseline. A representative trace was acquired from 9 traces. **(B)** The inset shows an enlargement of the positive component of the EOG highlighted with a circle, including the latency period following odorant stimulation. Rat olfactory epithelium was exposed to 1.6 mM of a standard odorant mixture at 25°C.

The electroolfactogram (EOG) is a summation of OSN generator potentials recorded extracellularly at the surface of the vertebrate olfactory epithelium (23, 24). While the well-understood excitatory pathway described above generates a negative extracellular charge and is the most common EOG representation of activated cellular response, many electrophysiological studies have reported positive voltage generation that are less understood (25–30). In our own EOG recordings, we frequently observe a small-amplitude positive peak preceding the depolarization event (Figure 1) that we aim to understand further. While olfactory cell excitation and signal transduction is a focal point in understanding olfactory processing, characterizing the full scope of inhibitory mechanisms present is also critical in understanding the odor stimulus-receptor relationship and how this may affect regulation of cellular response. Many investigators have attributed inhibitory currents observed in olfactory cells only to post-excitation negative feedback responsible for cell deactivation or related phenomena such as adaptation and desensitization (31–34). The unique nature of the inhibitory conductance discussed here is the timing of activation prior to depolarization and the seemingly obscure contexts in which it is observable.

This initial positive voltage transition was first described in early electrophysiological work by Ottoson (22) in frog and rabbit tissue, where it was characterized as an amplitude that is independent of odorant concentration but positively dependent on odorant temperature. Ottoson also observed positive transients when his electrode contacted nonolfactory tissue, and ultimately concluded that this was an artifact. In subsequent years, varied interpretations have emerged from experimental efforts to understand the nature of the positive transients in EOG readings. Takagi et al. (35–38) described the presence of positive and negative electrical components as “on” or “off” signals that were functionally relevant due to differential elicitation by different odorants. In a frog olfactory tissue model, Gesteland et al. (24) recorded EOG responses to a variety of odorants and control stimuli such as odor-free air puffs, distilled water vapor, or mechanical disturbance at changing temperatures. They observed varying effects on presence, duration, amplitude, and fatigue of each EOG component with different odorants, and suggested the preceding positive conductance had functional significance in olfactory transduction specific to certain odorants or odorant categories. However, with the understanding that the EOG is a complex electrical summation of many neurons, the observed positive conductance was not initially attributed to single cell activity. The following year, Gesteland et al. (39) refined this interpretation after repeated experimental observations, confirming that the EOG is comprised of two opposing processes representing functional activity. One process driving the potential in a positive direction was identified as inhibitory in function, and the other was related to the excitatory function of olfactory receptor response. However, the mechanism and nature of the inhibitory positive EOG component was still unclear. In later years, Getchell et al. (40) attempted to understand this relationship by designing an experimental system and theoretical model to separate the opposing electrical components in frogs. His results confirmed that different biological processes generated negative and positive voltage transients within the olfactory epithelium, but he did not identify the source or characterize the mechanism behind this relationship. After much breakthrough olfactory research identifying olfactory receptors as being in the family of G-protein coupled receptors (6), further characterization of the components along this signaling pathway allowed for more specific experimental approaches.

Through genetic mutation, Brunet et al. (41) conducted experiments with mice lacking functional olfactory cyclic nucleotide-gated ion channels and observed no negative component in odorant-evoked EOG which can be attributed to the lack of OSN depolarization. However, interestingly, a prominent positive EOG peak was still observed. At this time, the authors attributed this positive conductance to the surrounding non-neuronal supporting cells in the OE. Further mutation studies included a mouse model lacking functional olfactory G-proteins (42) and olfactory CNG ion channels, each thought to be responsible for the depolarization EOG component. Upon challenge with trimethylamine odor, they observed similar results of a positive EOG component in the absence of a negative depolarization peak. These authors also concluded that the positive current was of non-neuronal origin and most likely an artifact. In much later human olfactory studies, Lapid and Hummel (20) also suggested that the observable positive EOG transient preceding negative responses might be unrelated to excitatory activation and possibly linked to earlier mechanisms of response regulation. The nature and mechanism of preceding inhibitory currents are still not well understood due to species variation among the literature. However, along this timeline the conductance was identified as a carrier of potassium (K^+^) ions which prompted further K^+^-targeted methods of study.

In 1986, Trotier et al. (25) analyzed membrane currents in salamander olfactory receptor cells and found an inhibitory outward potassium current appearing before depolarization that was abolished in the presence of the K^+^-blocker, tetraethylammonium (TEA). Interestingly, when this inhibitory peak was blocked, the resulting depolarization current noticeably decreased. The author suggested a possible functional role of this inhibitory channel as a safeguard against excessive membrane depolarization in the presence of large odorant concentrations. McClintock et al. (43) discussed the presence of depolarizing and hyperpolarizing potentials in lobster olfactory receptor cells, also suggesting that K^+^ was the carrier of the odor-activated positive conductance and that the localization of these opposing currents to the same cell implicated regulatory significance. In later work by the same investigators, potassium channel blocking agents reportedly inhibited odor-evoked hyperpolarization as well as depolarization in a dose-dependent manner (44). They suggested the activation of this channel occurred at the receptor site prior to initiation of the action potential, though maintained the possible presence of additional potassium channels activated by downstream events that together formed a robust and complex regulatory mechanism. While these investigations have demonstrated the occurrence of pre-excitation inhibition, there has yet to be strong agreement on this phenomenon and demonstrations have been limited to invertebrates or non-mammalian vertebrates. As most physiological inhibition involves negative feedback mechanisms, it was logical to attribute the inhibitory currents only to response deactivating events, which have been thoroughly explored though not fully elucidated.

In 1995, Morales et al. (45) suggested that the inhibitory potassium current in olfactory neurons of toads was diminished by another K^+^-blocker, charybdotoxin. Despite the experimental data of Trotier (25) and McClintock (44) demonstrating its appearance prior to excitatory ion currents, the idea of direct odor activation of inhibitory potassium current was rejected due to known presence of calcium-sensitive potassium channels. Immunohistochemical analysis of the rat olfactory ciliary membrane (46) identified an −116 kDa protein that was proposed to be responsible for the potassium inhibitory currents found in rat and toad cilia preparations, and further analysis identified four different types of Ca^2+^-dependent potassium channels. Castillo et al. (47) further characterized these Ca^2+^-dependent K^+^ channels by reconstitution of rat olfactory cilia in a planar phospholipid membrane.

The controversial inhibitory pathway was explored with pharmacological mediators of CNG channel opening and cAMP concentration. Delay et al. (30) observed either excitatory or inhibitory responses in different neurons in the presence of increased cAMP and did not observe either conductance in transgenic mice lacking one subunit of the CNG channel, ultimately concluding that the inhibitory potassium current was CNG channel-mediated. Using a cyclic nucleotide-gated channel blocker and adenylyl cyclase (AC) inhibitor to both prevent production of cAMP and the opening of its target ion channel, Madrid et al. (48) found a reduction of odor-evoked Ca^2+^-dependent K^+^ channel conductance in isolated toad olfactory neurons. They concluded that a cAMP cascade is responsible for initiating the inhibitory Ca^2+^-dependent K^+^ current and shared the previously described suggestion made 10 years earlier by Morales that, during excitation, Ca^2+^ ions enter cilia through cAMP-promoted channel opening and subsequently activate inhibitory potassium channels.

These conclusions, however, contradicted experimental data showing an inhibitory EOG component preceding excitatory EOG transients (22, 24, 25, 39). Moreover, this conclusion contradicted the experimental results of Belluscio et al. (42) and Brunet et al. (41) who showed that the olfactory epithelium of a mutant mouse lacking known excitatory components, including functional G-proteins and cAMP-gated ion channels, produced inhibitory EOG transients with no excitatory EOG component. Many studies have demonstrated the presence and partial structural characterization of a subset of calcium-activated potassium channels that are thought to be associated with negative feedback mechanisms of cellular deactivation (27, 46, 49–51), however, the variation in molecular sensitivity and localization reported thus far suggests an incomplete understanding that does not fully exclude the possibility of odor-activated inhibitory currents.

Although the positive transient of the electroolfactogram has been widely considered throughout a long history of experimental investigation, inconsistent or possibly incomplete results have led to conflicting conclusions. Therefore, the mechanisms driving this phenomenon, nature of origin, and functional role in olfaction remains obscure. In the present study, we experimentally studied the functional and temporal involvement of this ionic current in rat olfactory epithelium by measuring EOG responses under varied applications of potassium channel inhibitors (4-Aminopyridine, charybdotoxin, iberiotoxin, and apamin) and an enzymatic inhibitor (3-isobutyl-1-methylxanthine) to generate downstream activation without odor stimulation. Many types of potassium channels are present in biological systems that are primarily involved in maintenance of cellular membrane potential and are generally classified into voltage-gated (K_V_) channels, Ca^2+^-activated (K_Ca_) large or small conductance channels, inward rectifier current channels, and leak channels (52). Single channel current recordings have characterized the pharmacological selectivity of blocking agents for certain channel types, so we selected a variety of potassium blocking agents for this work. While 4-Aminopyridine (4-AP), Charybdotoxin (ChTX), and Iberiotoxin (IbTX) broadly effect voltage-gated potassium channels (53–56), Apamin is highly selective for small conductance calcium-activated potassium channels (57, 58). IBMX inhibits the activity of phosphodiesterase which is responsible for breakdown of cAMP, thereby increasing its concentration and subsequent opening of CNG-gated ion channels. Together, these help us understand both the effects of potassium channel activity on odor responses as well as localize where along the transduction pathway this interaction occurs.

In the presence or absence of potassium conductance, we assessed both odor-evoked activity and non-odor, downstream-activated signal responses. Upon brief odor stimulation, we observed a consistent inhibitory current prior to the action potential that was diminished in a dose-dependent manner with the application of 3 out of 4 selected potassium channel blockers (4-Aminopyridine, charybdotoxin, and iberiotoxin). Furthermore, reduced peak amplitude of the excitatory negative current was observed with potassium channel blocking. When downstream activation of the GPCR transduction cascade was induced in the absence of odor stimulus, we observed an excitatory negative current that was not affected by administration of potassium blocking agents. Following non-odor excitation, we challenged olfactory receptors with zinc nanoparticles which have been previously shown to enhance olfactory EOG responses when delivered with odorant stimuli (59–61) with and without potassium inhibition, and observed a similar diminished positive and negative EOG amplitude. Here, we suggest the functional and temporal involvement of inhibitory potassium conductance in olfactory sensory neurons upon odor stimulation.

## Materials and methods

All procedures were performed in accordance with relevant guidelines and regulations.

### Animals

The animal protocol was approved by the Auburn University Institutional Animal Care and Use Committee (AU IACUC). Adult male Sprague–Dawley rats (Envigo, Dublin, VA) weighing −300 g were the approved animal model.

### Electrophysiological recordings

Electrophysiological methods used here were previously described in our earlier publications (59). Briefly, the rat olfactory epithelium was dissected and positioned in a perfusion chamber such that the basal parts were immersed in buffer solution (137 mM NaCl, 5.3 mM KCl, 4.2 mM NaHCO_3_, 0.4 mM KH_2_PO_4_, 3.4 mM Na_2_HPO_4_, 5.6 mM D-glucose, 0.8 mM MgSO_4_, and 1.2 mM CaCl_2_ at pH 7.4), whereas the extracellular-facing cilia were positioned at the water/air interface. EOG recording glass electrodes were linked through the Ag/AgCl wire to an amplifier to record signals from the olfactory epithelium. After establishing the connection between the electrode and olfactory epithelium, an air pulse of the odorant mixture was directed toward the epithelial surface, and a continuous electrical signal was recorded as a function of time. Odor-activated voltage responses amplified (MultiClamp 700A Amplifier, Molecular Devices) and relayed to the recording monitor as a digitized EOG measurement through an analog-to-digital filter at 0–5 kHz and digital converter (DigiData 1322A, Axon Instruments). Data were recorded at a sampling frequency of 10 kHz. Data acquisition, storage, and subsequent analysis were carried out using pCLAMP software (Axon Instruments) and exported in ASCII format for further analyses. The recording chamber was enclosed in a grounded Faraday box on a vibration isolation table (GS-34 Newport). The odorant vapor was produced by a homemade olfactometer (62) that was used for the precise computer-controlled delivery of predetermined quantities of odorants over a programmed time interval. A pulse of positive pressure drove the odorant into a glass nozzle directed at the olfactory epithelium. The mean value of the relative change of 2 consecutive EOG peaks stimulated by the same odorant pulse (|ΔV/V|) was 4.5% (0.045 ± 0.010 [standard error (SE)], 10 recordings, 0.75 ± 0.2% min, respectively. Assuming no drift, the sample SEs were a measure of repeatability. In 30-min tests, the SEs were less than 3% for EOG recordings.

### Odorants and pathway-mediators

#### Odorant delivery

An odorant mixture of ethyl butyrate, eugenol, and (+) and (−) carvone in water was prepared with a vortex mixer at 1.6 mM each, and stored in a dark glass container at 278 K (5°C) (59). The odorant vapor was produced by a homemade olfactometer (62) that was used for the precise computer-controlled delivery of predetermined quantities of odorants over a programmed time interval. For stimulation purposes, a 0.25 s pulse of the odorant mixture was formed by a computer-controlled Pneumatic PicoPump PV800 (World Precision Instruments, Sarasota, FL, USA). The automatic computer routine was composed of 0.25-s pulses at 20- and 60-s intervals. One series of 10 pulses at 20-s intervals constituted one “Recording”. Thus, one recording consisting of 10 response traces had a duration of 200 s. These recordings were repeated as many times as needed to cover a desirable number of pulses and duration for a single experiment. A single experiment contained recordings obtained from a single sample of olfactory epithelium. A pulse of positive pressure drove the odorant from the backspace of a bottle into a glass nozzle directed at the olfactory epithelium. Because all odorants utilized in our studies have a very low water/air partition coefficient (−10^−4^), the concentrations delivered to the olfactory tissue are quite low. The concentration of odorants in the headspace is estimated in parts per billion. The concentration of Eugenol in head space, for example, can be estimated using the Amoore-Buttery equation (63):

$$K_{aw}=\left(\left(\frac{55.5}{S-0.0555}\right)\times M+1\right)\times P\times 0.97\times 10^{-6}$$

Where K_aw_, partition coefficient, S is solubility g/L of the pure odorant at 25°C, P is vapor pressure in mm Hg, and M is molecular weight. For Eugenol, we have *P* = 0.0226 mm Hg; S = 2.47 g/L; M = 164.2 g/mol. According to the Amoore-Buttery equation, *K_aw_* = 8.08 × 10^−5^. This value of K_aw_ for Eugenol agrees well with that obtain experimentally (64). Thus, the concentration of Eugenol in the bottle head space (and therefore delivered to the olfactory epithelium) is calculated as

$$\begin{array}{rl} & C_{h}\;=\;K_{aw\;}\;\times \;C_{b}\;=\;8.08\;\;\times \;10^{-5}\;\;\times \;1.6\;\;\times \;10^{-3}\\ & \;M\;=\;1.3\;\;\times \;10^{-7}M\;=\;13|\mu M, \end{array}$$

where *C_h_* is a bottle head space concentration and *C_b_* is balk concentration in liquid.

#### K^+^-ion channel inhibition

K^+^-channel blockers including 4-aminopyridine (2.5 nM), charybdotoxin (0.4 nM), iberiotoxin (0.4 nM), and apamin (0.1 nM) were dissolved in buffer solution and delivered to the olfactory epithelium by small droplets, with the estimated concentration distribution calculated by the following stepwise equations. A droplet of the blocker-water solution was released from a micropipette position at 4 mm above the contact point of the EOG measuring electrode. The droplet reached the water surface in −16 ms and a maximum spread factor was reached within a few more milliseconds. This was calculated by the following equation (65):

$$\zeta =\frac{D_{max}}{D_{0}}=\sqrt{\frac{We+12}{3(1-cos\theta_{a})+4\left(\frac{We}{\sqrt{Re}}\right)}}\;\;\;\;$$

Weber number, $We=\rho v^{2}D_{0}/\sigma$, Reynolds number, $Re=\rho vD_{0}/\eta$, *Ρ* – density, *ν* – droplet velocity, D_0_ – initial droplet diameter, D*_max_* –maximum spread diameter, *σ* – surface tension, *η*- dynamic viscosity, *θ*_a_ – dynamic contact angle (66).

For a droplet of D = 0.216 mm, released from 4 mm of the water surface, the estimated spread factor using the above equation was found equal to 3.7 with a maximum spread of D*_max_* = 0.8 cm. The gradual diffusion of the blocker and resulting concentration distribution can also be calculated by the following equation (67):

$$c=\frac{M}{\sqrt{4\pi Dt}}\;$$

Where *M* is a surface concentration of blocker molecules in grams per square centimeter, *D* is a diffusion coefficient of the blocker molecules in water in cm^2^/s, and t is time in s.

Experiments with blockers were carried out in the following order: (1) response to water vapor (control), (2) response to odorant alone, (3) response to a mixture of odorant and blocker, and (4) five replicate responses to odorant alone following blocker application.

#### Downstream signal activation

Membrane excitation in the absence of odor stimulation was generated with a membrane-permeable phosphodiesterase inhibitor, 3-isobutyl-1-methylxanthine (IBMX), which prevents the enzymatic breakdown of the second messenger cyclic adenosine monophosphate (cAMP), thus increasing its concentration and downstream activation of cyclic nucleotide gated channels through which Na^+^ and Ca^2+^ enter the cell to initiate membrane depolarization. This chemical agent was delivered through a specially designed multi-pipette system mounted on the Soma MX1100 R High-Precision Micromanipulator for simultaneous application of buffer and IBMX onto the few hundred micron-diameter epithelial space as described previously (60). The pipette tips containing either buffer alone or 30 µM IBMX dissolved in buffer contacted the OE, and delivered as a 0.25 s pulse.

Odorants, 4-Aminopyridine, charybdotoxin, iberiotoxin, apamin, and IBMX were obtained from Sigma-Aldrich.

### Statistical analysis

Data averaging, curve fitting, and graph plotting were performed using Origin 2019 (Northampton, MA, USA) and Microsoft Excel 2010. Negative EOG data comparisons were carried out using one-way ANOVA followed by Tukey's multiple comparison test. Additionally, linear correlation analysis was used to align relative EOG amplitudes with concentrations of odorant and the area under the peak.

## Results

### Odor-evoked positive EOG currents that were sensitive to potassium blocking agents preceded and affected the relative amplitudes of excitatory negative EOG currents

**Figure 2:**
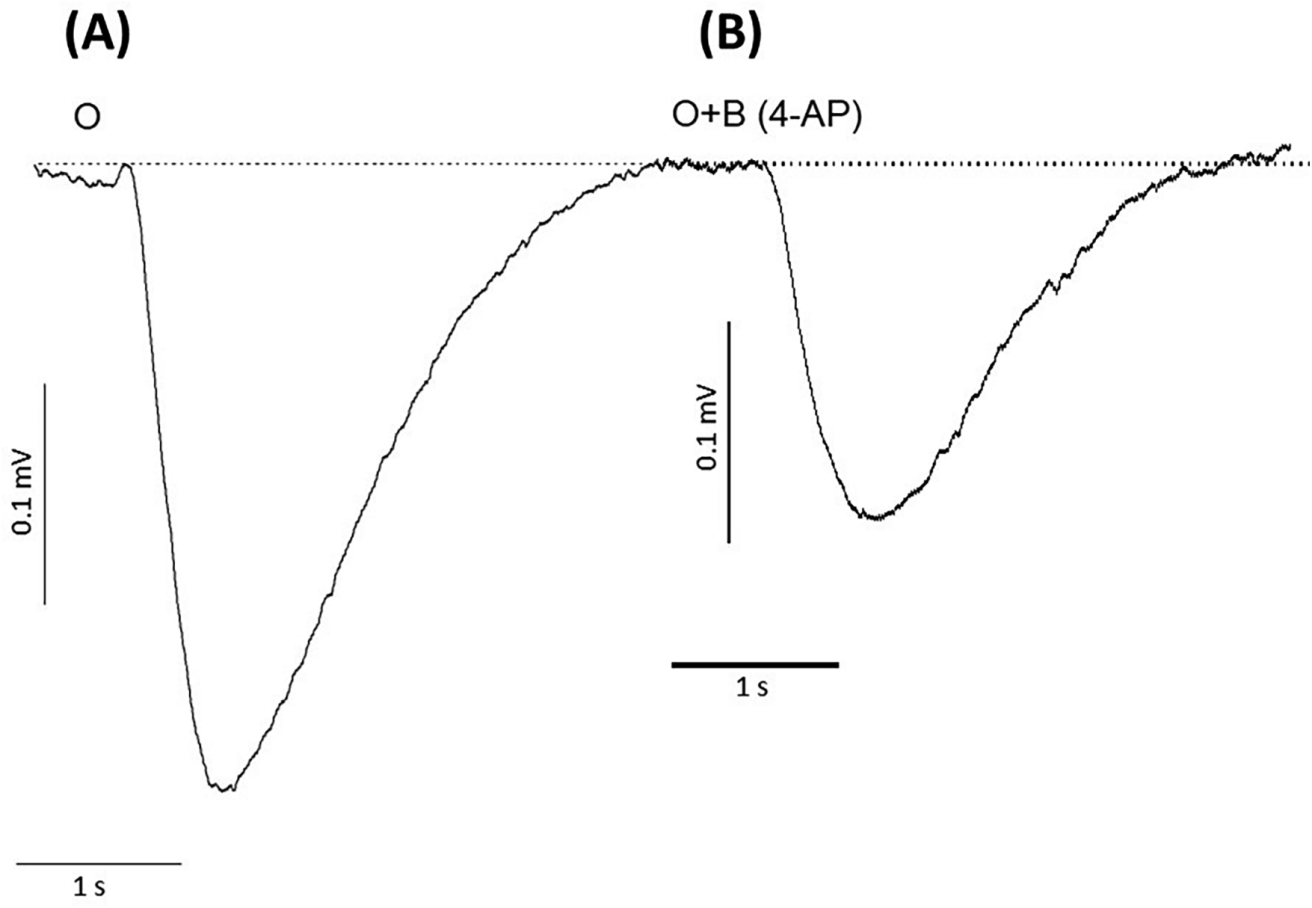
Responses of negative EOG recorded from rat olfactory epithelium in the absence or presence of the potassium ion channel blocker, 4-aminopyridine (4-AP). **(A)** EOG recording induced by 1.6 mM odorant (O), **(B)** EOG induced by odorant with 2.5 nM 4-aminopyridine (O + B 4-AP). Odorant concentration = 1.6 mM, Latency time = −160 ms, Stimuli duration = 0.25 s, Vertical bars = 0.1 mV, Horizontal bars = 1 s, Temperature = 25°C.

**Figure 3:**
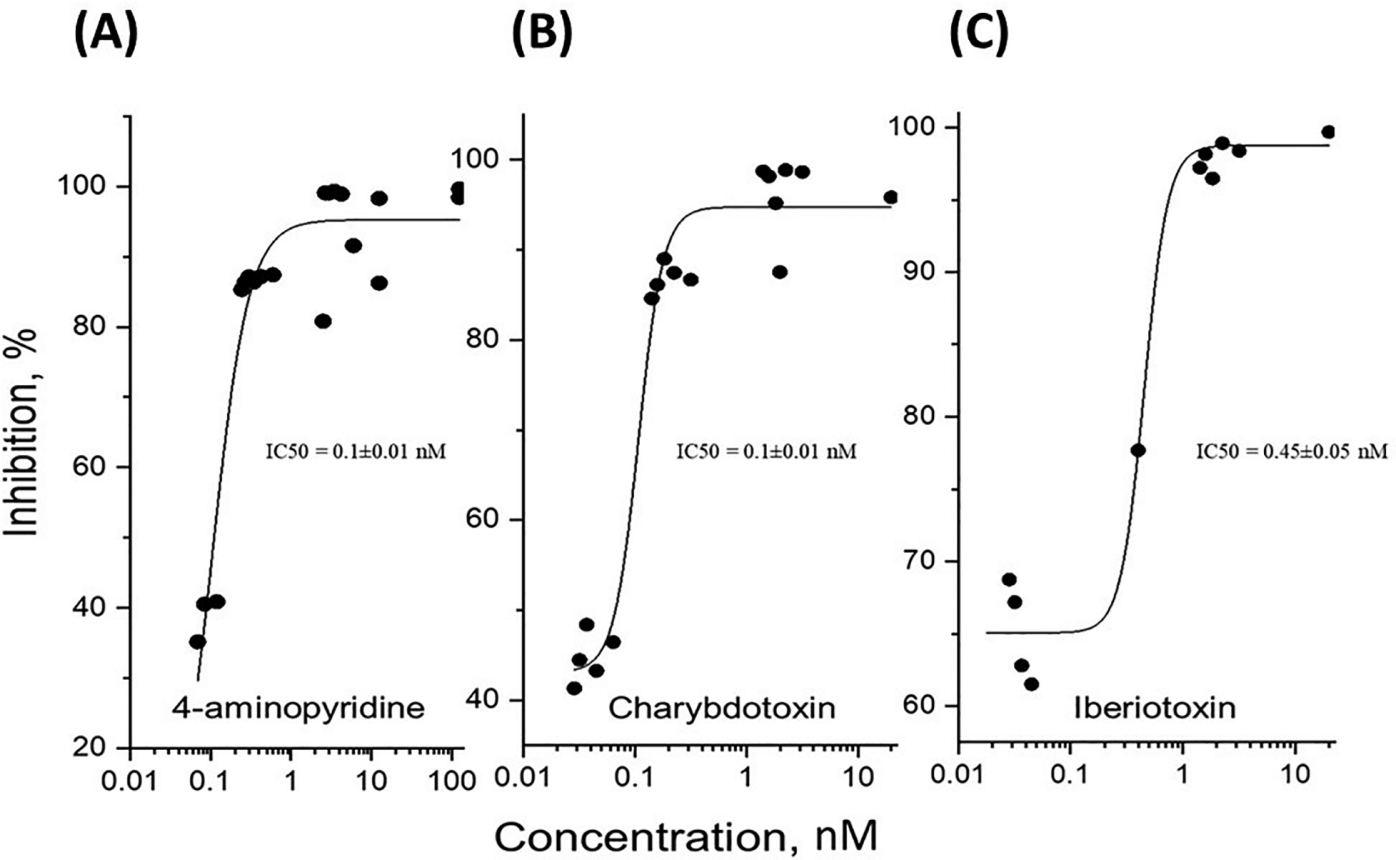
Dose-dependent inhibition of potassium channels expressed as the ratio of relative signal amplitudes. The experimental data points represent the relative odor-evoked negative EOG amplitudes with or without a blocker, as the ratio of (O + B)/O expressed as percentage of inhibition for increasing blocker concentration. The data points were fitted to sigmoidal curves according to the logistic equation, *Inh = Inh_max_ + (Inh_min_-Inh_max_)/[1 + (Inh)/IC50]^p^)*, where Inh, Inh_max_, and Inh_min_ are the inhibition, the maximal, and minimal inhibition, respectively. IC50 is the half maximal effective concentration of inhibitor, and p is a constant related to the slope of the sigmoidal curve. **(A)** The best fit for 4-aminopyridine gave the following parameters: Inh_max_ = 95.2 ± 2.0 (SE) %, IC50 = 0.1 ± 0.01 (SE) nM, *p* = 1.9 ± 0.3 (SE), R^2^ = 0.97. **(B)** The best fit for charybdotoxin gave Inh_max_ = 94.8 ± 1.5 (SE) %, IC50 = 0.1 ± 0.01 nM, *p* = 3.9 ± 1.1 (SE), R^2^ = 0.97. **(C)** Fitting the iberiotoxin dose-dependence gave Inh_max_ = 98.8 ± 1.5 (SE) %, IC50 = 0.45 ± 0.05 (SE) nM, *p* = 4.1 ± 1.8 (SE), R^2^ = 0.98.

**Figure 4:**
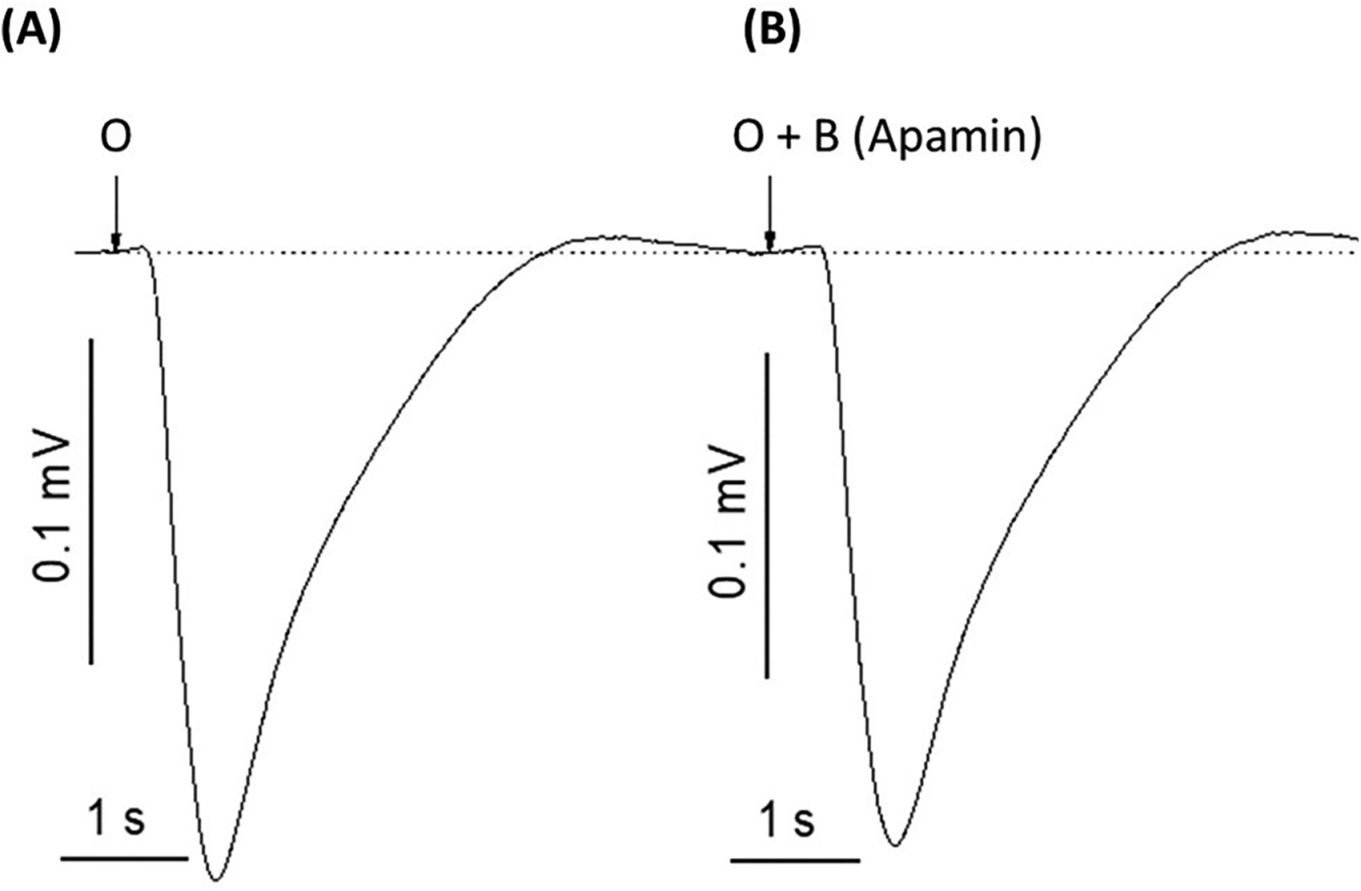
Apamin, a peptide neurotoxin found in bee venom known to selectively block Ca^2+^-activated K^+^ channels, does not affect odor-evoked EOG response. (**A**) EOG recording induced by 1.6 mM odorant (O). (**B**) EOG trace induced by 1.6 mM odorant and 0.01 nM of apamin (O + B-apamin). Representative traces out of 9 recordings. The experiment was replicated four times. Stimuli duration = 0.25 s, Vertical bars = 0.1 mV, Horizontal bars = 1 s, Temperature = 25°C.

To determine whether potassium ions are involved in the olfactory response to odorants, we exposed rat olfactory epithelium to a pulse of odorant mixture alone or with potassium ion channel blocker, 4-aminopyridine, as described in other work (68). When tissue was exposed to a pulse of odorant without a blocking agent, the EOG showed an excitatory negative peak (Figure 2A). When exposed to the mixture of odorant and 4-aminopyridine, the EOG response did not show a positive peak but did display a considerable reduction in the negative peak (Figure 2B). Alternative potassium channel inhibitors, charybdotoxin, iberiotoxin, and apamin, were administered in a similar manner. Odor-evoked excitatory peaks were also reduced in a dose-dependent manner by charybdotoxin and iberiotoxin, together represented by sigmoidal growth curves of percent inhibition as a function of concentration (Figures 3A–C). The calculated IC50 values for the three effective potassium blockers ranged from 0.1 nM–0.45 nM and maximum inhibition values between 94.8%–98.8%. However, there was no observed decrease with apamin (Figure 4) which has shown to selectively block Ca^2+^-activated K^+^ channel. A one-way ANOVA was performed to compare the effect of 4-aminoperidine on odor-evoked negative EOG peaks, revealing a statistically significant difference in EOG depolarization [F(1, 6) = 28.8, *p* = 0.0017]. Tukey's HSD Test for multiple comparisons also found that the mean value of the negative EOG was significantly different between the odor-evoked excitatory response when potassium channel activity was blocked compared to unblocked [*p* = 0.0017, 95% C.I. = (0.041, 0.109)].

### A preceding positive potassium channel current was not observed with downstream activation of depolarization

**Figure 5:**
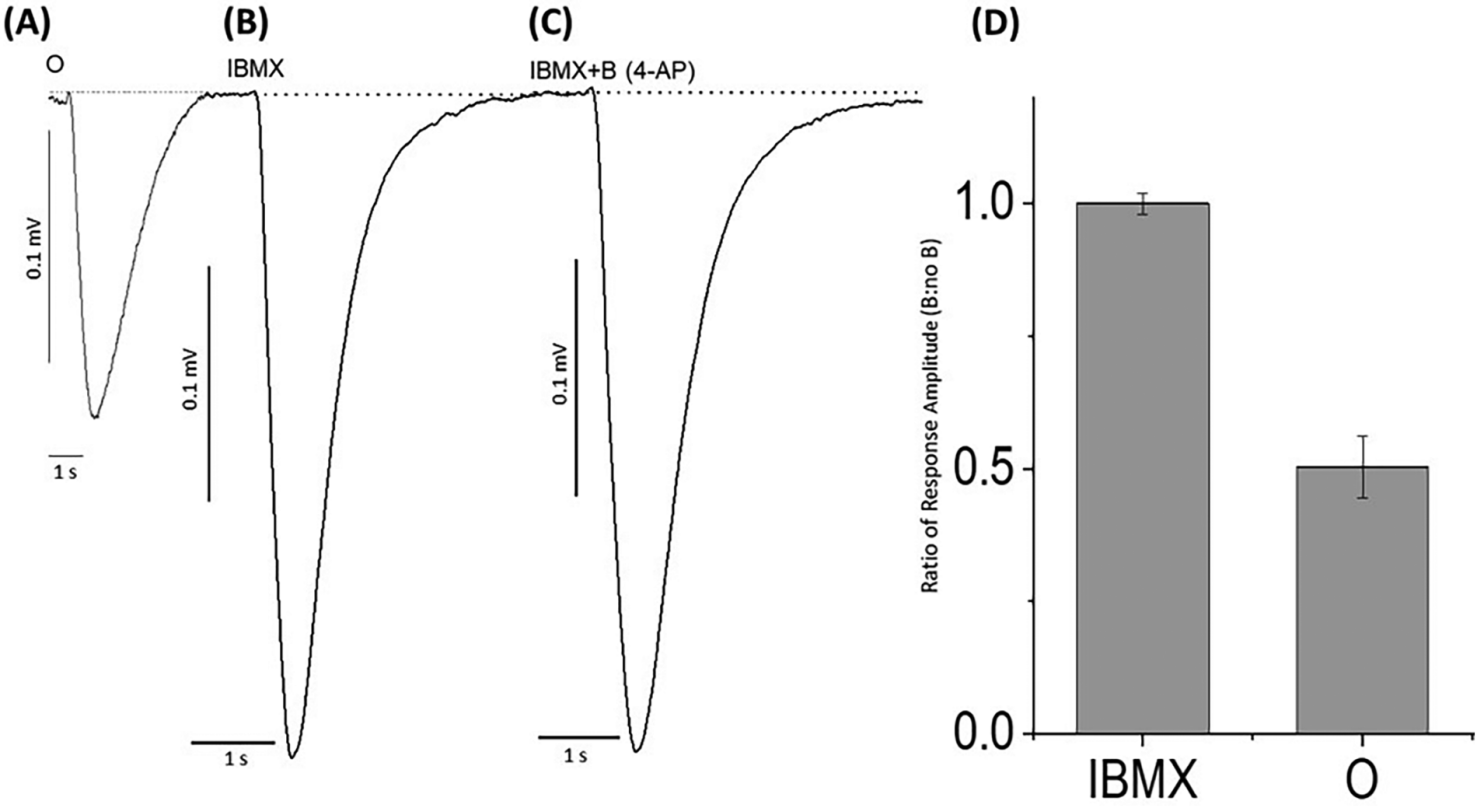
EOG responses recorded from odor-stimulated and phosphodiesterase inhibitor (IBMX)-stimulated rat olfactory epithelium with or without potassium ion channel blocker, 4-aminopyridine. **(A)** EOG elicited by 1.6 mM odorant (O) (control), **(B)** EOG responses induced by 30 µM IBMX without a blocker, **(C)** EOG responses induced by 30 µM IBMX with 2.5 nM 4-aminopyridine (IBMX + B). **(D)** The relative amplitude induced by (IBMX + B)/IBMX was equal to 1.0 ± 0.02. Similarly, relative amplitudes (O + B)/O = 0.50 ± 0.06. The data were obtained from 4 experiments. Odorant concentration = 1.6 mM, Latency time = −160 ms, Stimuli duration = 0.25 s, Vertical bars = 0.1 mV, Horizontal bars = 1 s, Temperature = 25°C.

These experiments indicated that the potassium blocker, 4-aminopyridine, caused an evident reduction in the negative EOG peak generated by the Ca^2+^, Na^+^, and Cl^−^ ion channels in the cAMP cascade that was restored upon removal of the blocking agent. This suggested a downstream mediation in which the potassium channel conductance is involved in depolarizing activity, so to examine this conclusion, we excited the cAMP cascade by the membrane-permeable phosphodiesterase inhibitor 3-isobutyl-1-methylxanthine (IBMX) (69). When the enzymatic decomposition of cAMP was eliminated by IBMX, the increased cAMP produced EOG signals without the participation of olfactory receptors. Figure 5 demonstrates that in this case, EOGs evoked by IBMX and IBMX with 4-aminopyridine were identical. This suggested that the potassium blocker, 4-aminopyridine, blocked potassium ions outside the cAMP-activated calcium-dependent channels. A one-way ANOVA was performed to compare the effect of 4-aminopyridine on the negative EOG excited by IBMX. A one-way ANOVA revealed that there was a statistically significant difference in the negative EOG excited by IBMX between at least two groups [F(2, 9) = 15.1, *p* = 0.013]. There was no statistically significant difference between the negative EOG excited by IBMX and EOG excited by IBMX with of 4-aminoperidine (*p* = 0.97).

### Odor-evoked negative EOG responses that were enhanced with the addition of zinc nanoparticles were also diminished with potassium channel blocking and partially restored upon removal of blockers

**Figure 6:**
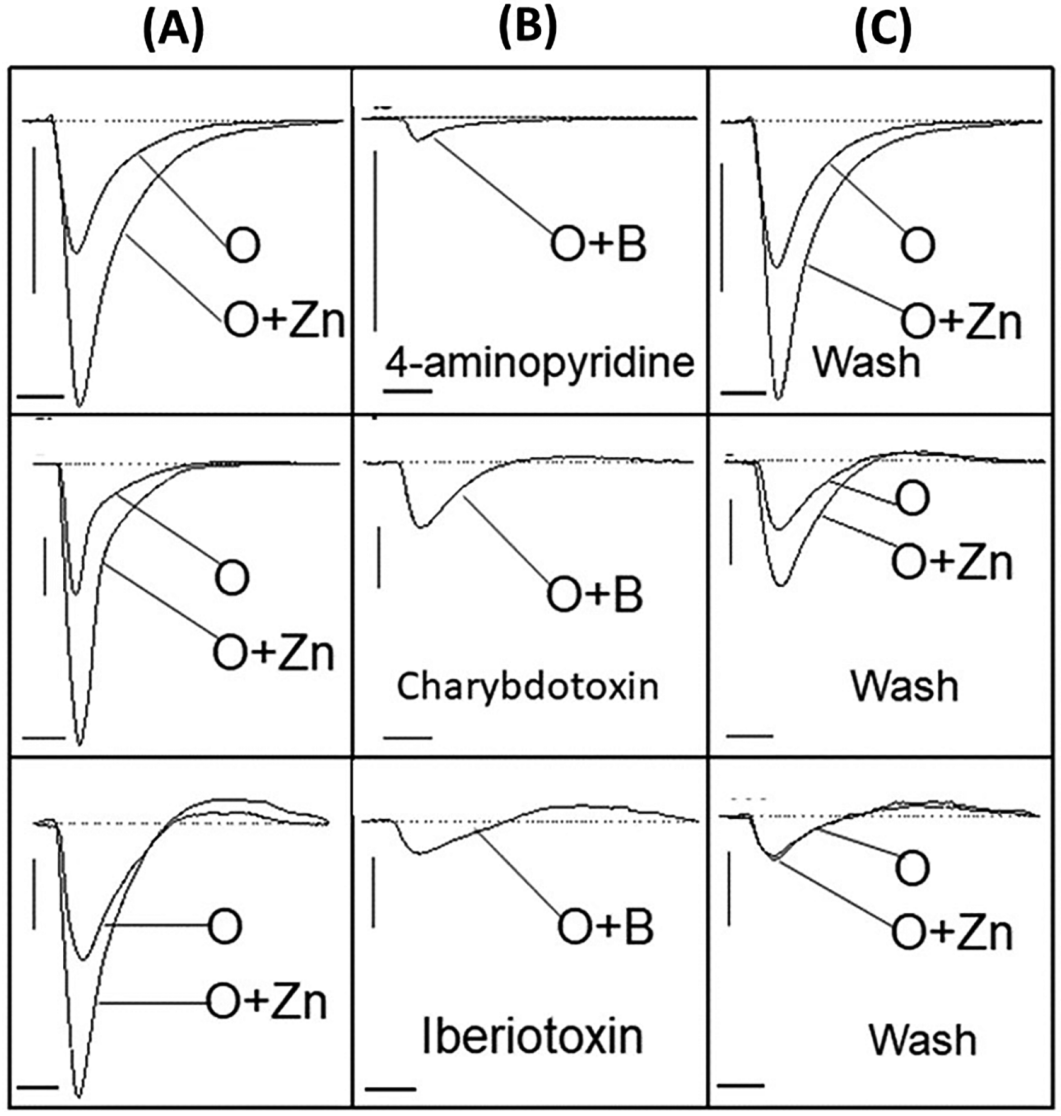
Restoring potassium current by washing recovers the negative EOG. Three channel blockers were delivered with 1.6 mM odorant (O) and 20 pM enhancing agent, zinc nanoparticles (O + Zn), prior to washing with 2.5 ml buffer solution. **(A)** Vertical panel showing EOG recordings generated by odorant alone (O) and odorant with zinc nanoparticles (O + Zn), **(B)** Vertical panel showing EOG traces evoked by 1.6 mM odorant (O) together with potassium blockers (O + B) – 2.5 nM 4-aminopyridine, 0.4 nM iberiotoxin, and 0.4 nM charybdotoxin, **(C)** Vertical panel showing restored EOG signals after washing each blocker away from respective tissue with 2.5 ml buffer solution. Stimuli duration = 0.25 s, Vertical bars = 0.1 mV, Horizontal bars = 1 s, Temperature = 25°C.

After evaluating the excitatory response of non-odor stimuli, we further assessed odor-evoked response when challenged with potassium blocking agents in the presence of an enhancing agent (zinc nanoparticles). Here, an odorant mixture was applied along with an odor-enhancing agent, zinc nanoparticles (59) (Figure 6A), and a prominent negative peak was observed that was diminished when combined with 2.5 nM of 4-aminopyridine, 0.4 nM charybdotoxin, and 0.4 nM of iberiotoxin, respectively (Figure 6B). Following removal with 2.5 ml of washing buffer and subsequent odor stimulus with or without zinc nanoparticles, the negative EOG peaks were recovered to a varying degree (Figure 6C), with the most complete restoration of tissue challenged with 4-aminopyradine blocker.

## Discussion

Here, we found that a positive EOG current sensitive to potassium blocking agents was strongly correlated with the amplitude of the negative EOG peak representing a physiological response to odorant. Although the negative EOG peak is conventionally attributed to Ca^2+^ and Cl^−^ ion channels (16, 17, 70), we found that the potassium ion channel blocker, 4-aminopyridine, significantly reduced the odor-evoked amplitude of the negative electroolfactogram. This suggested likely participation of signal transduction mediation outside the cAMP amplification complex. Supporting this, we found that the potassium channel blocker did not affect the EOG responses excited without odor-stimulation of olfactory receptors by using enzymatic blocker, IBMX. The EOG negative peak was also inhibited by charybdotoxin and iberiotoxin but was not sensitive to apamin. The pharmacological profiles and washout kinetics of potassium channel blockers applied to EOG excited by odorants in our experiments were similar to those observed in the blockade of potassium channels in olfactory neurons and cilia of toads and rats (45, 47, 48, 71, 72). It should be noted that 4-aminopyridine, charybdotoxin, and iberiotoxin inhibit broad spectrum voltage-gated K^+^ channels, while apamin is highly selective for Ca^2+^-mediated channels (57). Mechanisms of potassium channel modulation differ depending on the molecular nature of the compound and exposing these differences helps to characterize structure and function of channels responsible for a given potassium ion conductance. Blocking agents can either inhibit ion flux by binding to intracellular or extracellular domains of the channel pore or mediate channel gating through voltage-sensor binding. Binding affinity, strength, and localization to either intra- or extracellular domains all influence selectivity for potassium channel inhibition and clearance (73) and may explain the differences we observed in signal recovery following buffer washout.

There are also structural considerations relevant to the relationship between potassium channels and mode of activation. The idea of the olfactory receptor-site potassium channel is also supported by structural similarity of the transmembrane portion (four transmembrane helix – loop – transmembrane helix elements) of potassium channels and oligomers of G-protein coupled olfactory receptors (74). The x-ray structure of the full-length potassium channel of *streptomyces A* (KcsA) was also determined, offering further insight into the intracellular domain in both inactive and active states (75).In 1983, Vodyanoy and Murphy (76) reconstituted a potassium ion channel from rat olfactory epithelium in the planar phospholipid membrane and observed activation of the single channel of 62 pS upon stimulation with diethyl sulfide that was blocked by 4-aminopyridine. Potassium ion channels activated by odorants and sensitive to charybdotoxin and other K^+^ channel blockers were later found in toad and rat dissociated olfactory neurons by the whole cell patch clamp technique (45, 71, 72) single channel recording from olfactory cilia (46, 77), and reconstituted in planar lipid bilayers (47).

Overall, our results suggest involvement of an odorant-activated positive potassium channel conductance that precedes and mediates the amplitude of depolarization. While the hyperpolarization event has been traditionally described as inhibitory in nature, it does appear to have an excitatory effect as its removal causes diminished response strength. Increased excitability elicited by inhibitory stimuli has been described in different cells of the mammalian nervous system as a “post-inhibitory rebound”, but the mechanism is not yet established (78, 79). In a 1998 literature review of olfactory transduction pathways and the chronological discoveries that shaped the more divisive topics in the field, Schild and Restrepo (80) suggested that the varied findings across vertebrate and invertebrate experimental models indicates mechanistic diversity in the olfactory system. Rather than a universal mechanism of cellular excitation and return to baseline for every OSN, there is more likely a combination of suppressive and excitatory pathways on individual neurons and perhaps multiple excitatory fine-tuning mechanisms present which could be functionally significant in the discrimination and coding of the vast array of chemical odorants. These authors developed a working model of dual olfactory transduction pathways in OSN of spiny lobster which involves an odorant-activated cAMP-mediated cascade that can be either inhibitory or excitatory. In the inhibitory cascade, cAMP directly gates K^+^ channels leading to hyperpolarization. These findings and theoretical models do not negate the widely accepted conclusion that a single receptor type is expressed on single neurons, because it is possible that different motifs of a single receptor type elicit separate pathways of molecular activity and ionic channels. Furthermore, some odorants may activate these alternative pathways simultaneously and contribute to the refinement of odor coding.

Our experimental results leave us with two critical questions and a platform for further investigation. First, why does blocking the potassium channel affect downstream depolarization when it is thought that K^+^ ions are not involved in nucleotide-triggered excitatory currents? Second, why is the inhibitory positive EOG peak inconsistently observed under special conditions, including elevated temperatures, with certain odorants, or in slightly damaged olfactory epithelium? Earlier work reporting the presence of pre-action potential inhibitory EOG conductance discussed the possible temperature and odorant concentration dependency of the phenomena, so this is a critical assessment for further testing. While continued demonstration of potassium-mediated olfactory responses and their place along the transduction cascade must be further analyzed, it is possible that there are additional inhibitory mechanisms at play that are initiated both by negative feedback of calcium and earlier in the initial binding events at the receptor level.

## Data Availability

The raw data supporting the conclusions of this article will be made available by the authors, without undue reservation.
